# Anti-*Acinetobacter baumannii* single-chain variable fragments provide therapeutic efficacy in an immunocompromised mouse pneumonia model

**DOI:** 10.1186/s12866-023-03080-9

**Published:** 2024-02-10

**Authors:** Eilnaz Basardeh, Somayeh Piri-Gavgani, Hamid Reza Moradi, Masoumeh Azizi, Parastoo Mirzabeigi, Farzaneh Nazari, Mostafa Ghanei, Fereidoun Mahboudi, Fatemeh Rahimi-Jamnani

**Affiliations:** 1 Department of Mycobacteriology and Pulmonary Research, Microbiology Research Center, Pasteur Institute of Iran, Tehran, Iran; 2 Department of Basic Sciences, School of Veterinary Medicine, Shiraz University, Shiraz, Iran; 3 Molecular Medicine Department, Biotechnology Research Center, Pasteur Institute of Iran, Tehran, Iran; 4 Department of Clinical Pharmacy and Pharmacoeconomics, Faculty of Pharmacy, Iran University of Medical Sciences, Tehran, Iran; 5 Chemical Injuries Research Center, Systems Biology and Poisoning Institute, Baqiyatallah University of Medical Sciences, Tehran, Iran; 6 Biotechnology Research Center, Pasteur Institute of Iran, Tehran, Iran

**Keywords:** *Acinetobacter baumannii*, Pneumonia, Immunotherapy, scFv, Bactericidal antibodies

## Abstract

**Background:**

The emergence of carbapenem-resistant and extensively drug-resistant (XDR) *Acinetobacter baumannii* as well as inadequate effective antibiotics calls for an urgent effort to find new antibacterial agents. The therapeutic efficacy of two human scFvs, EB211 and EB279, showing growth inhibitory activity against *A. baumannii* in *vitro*, was investigated in immunocompromised mice with *A. baumannii* pneumonia.

**Results:**

The data revealed that infected mice treated with EB211, EB279, and a combination of the two scFvs showed better survival, reduced bacterial load in the lungs, and no marked pathological abnormalities in the kidneys, liver, and lungs when compared to the control groups receiving normal saline or an irrelevant scFv.

**Conclusions:**

The results from this study suggest that the scFvs with direct growth inhibitory activity could offer promising results in the treatment of pneumonia caused by XDR *A. baumannii.*

## Background

The carbapenem-resistant *Acinetobacter baumannii* (CRAB) has become one of the most clinically critical pathogens in medical care due to its severe threat to human health [1, 2]. Nosocomial infections, including pneumonia, bloodstream infection, urinary tract infection, and skin, soft tissue and bone infections caused by CRAB particularly threaten newborns, immunocompromised individuals, and patients hospitalized at intensive care unit (ICU) [3–11]. In particular, CRAB pneumonia, which has a high in-hospital mortality rate of up to 46%, has received a great deal of attention [1, 12]. Antimicrobial resistance (AMR), ranking as one of the top ten health threats in the world, has severely compromised the treatment of CRAB infections, particularly its tendency to be extensively drug-resistant (XDR), only susceptible to limited antibiotics (e.g. colistin and tigecycline), and pan-resistant isolates that are resistant to all antibiotics [11, 13]. Accordingly, the World Health Organization (WHO) has classified CRAB as a critical priority pathogen (Priority 1) for which functional antibiotics capable of combating CRAB infections are urgently needed [13].

The use of immunotherapy to combat respiratory infections caused by particular pathogens can significantly improve the effectiveness of treatment and minimize the side effects resulted from antibiotics [14–16]. Monoclonal antibody (mAb) is considered the pronounced arm of immunotherapy due to its great ability in recognition and binding to particular antigens leading to a range of simple (e.g. blockade of target) to multifaceted events (e.g. antibody-dependent cellular cytotoxicity and complement-dependent cytotoxicity) [16–18]. Of note, mAbs can enhance the efficacy of existing antibiotics (e.g. additive or synergistic effect) and minimize the adverse effects of toxic antibiotics (e.g. colistin) [19], or improve drug delivery to target sites (e.g. DSTA4637S comprised of a monoclonal THIOMAB™ IgG1 specific to *S. aureus* β-*O*-linked *N*-acetylglucosamine conjugated with a rifamycin analogue [dmDNA31]) [20, 21]. In this regard, several studies developed mAbs targeting different sites of *A. baumannii* (e.g. capsule, outer membrane protein A (OmpA), and Omp34) [22–26]. Compared to mAbs, antibody fragments have been less explored but include benefits such as better accessibility to hidden epitopes, development of bispecific antibodies, and cost-effective routes of production [27, 28]. Among the various antibody fragments, single-chain variable fragments (scFvs) stand out due to their particular construct containing only the variable regions of both the heavy and light chains [27–30]. The scFv antibody fragments benefit from their small size, suitable binding ability, and low immunogenicity [28, 31, 32]. In particular, the emergence of bactericidal scFvs as new antimicrobial biotherapeutics, exhibiting significant growth inhibitory activity against some pathogens in vitro and in vivo [30, 33–39], has opened up a new window of hope in combating against health-threatening pathogens. Targeting bacterial cell membranes and causing the disintegration of the lipid bilayer which leads to cytoplasmic leakage and bacterial death are introduced the major mechanism of actions of bactericidal scFvs [30, 34, 40]. Several bactericidal scFvs have been developed against pathogens such as *A. baumannii* [40], *Pseudomonas aeruginosa* [34, 35], relapsing fever *Borrelia* [41], and *Staphylococcus aureus* [30, 33], some of which could provide protection in the mouse model of infection.

In the previous report, we identified the two fully human scFvs, EB211 and EB279, which exerted bactericidal activity against *A. baumannii* [40] by displacing Mg^2+^ and interrupting the integrity of the outer membrane (data not shown). As part of the present study, we first determined histopathologically whether EB211, EB279, and a cocktail of the two scFvs have undesirable effects on healthy mice. Afterwards, the protective activity of the scFv was assessed in immunocompromised mice with pneumonia caused by an XDR *A. baumannii* strain, either alone or in a combination with another scFv twice a day (BID) for three days.

## Results

### The non-toxic potential of EB211 and EB279 in healthy mice

**Fig. 1:**
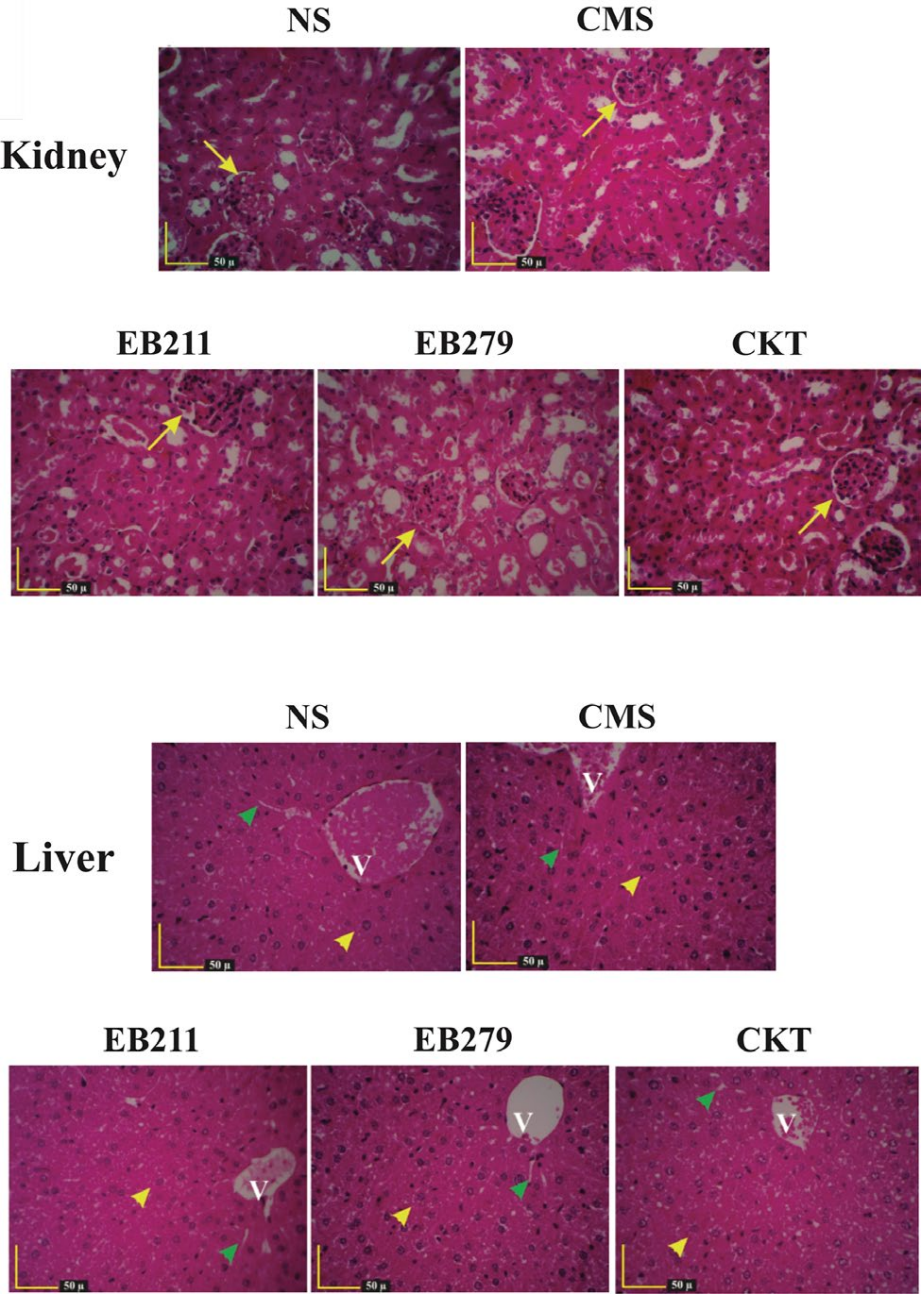
EB211 and EB279 caused no toxicity to the kidneys and liver of mice. No noticeable histopathological changes were observed in the kidneys and liver of mice injected intraperitoneally twice daily with EB211 (12 mg/kg), EB279 (12 mg/kg), or a cocktail of the two scFvs (CKT; 6 mg/kg of each) for 72 h, similar to mice injected intraperitoneally twice daily with colistimethate sodium (CMS; 30 mg/kg) or normal saline (NS). Green arrowheads: sinusoids, V: central venules, Yellow arrowheads: hepatocytes, Yellow arrows: renal tubule and renal corpuscle

The in vivo assessment of toxicity of EB211 and EB279 demonstrated no marked histopathological changes in the kidneys and liver of mice injected intraperitoneally twice daily with EB211 (12 mg/kg) and EB279 (12 mg/kg), and the combination of the EB211 and EB279 scFvs (6 mg/kg of each) for 72 h, similar to mice injected intraperitoneally twice daily with colistimethate sodium (CMS; 30 mg/kg) or normal saline (NS) (Fig. 1).

### Treatment effectiveness of EB211 and EB279 in immunocompromised mice with *A. baumannii* pneumonia

**Fig. 2:**
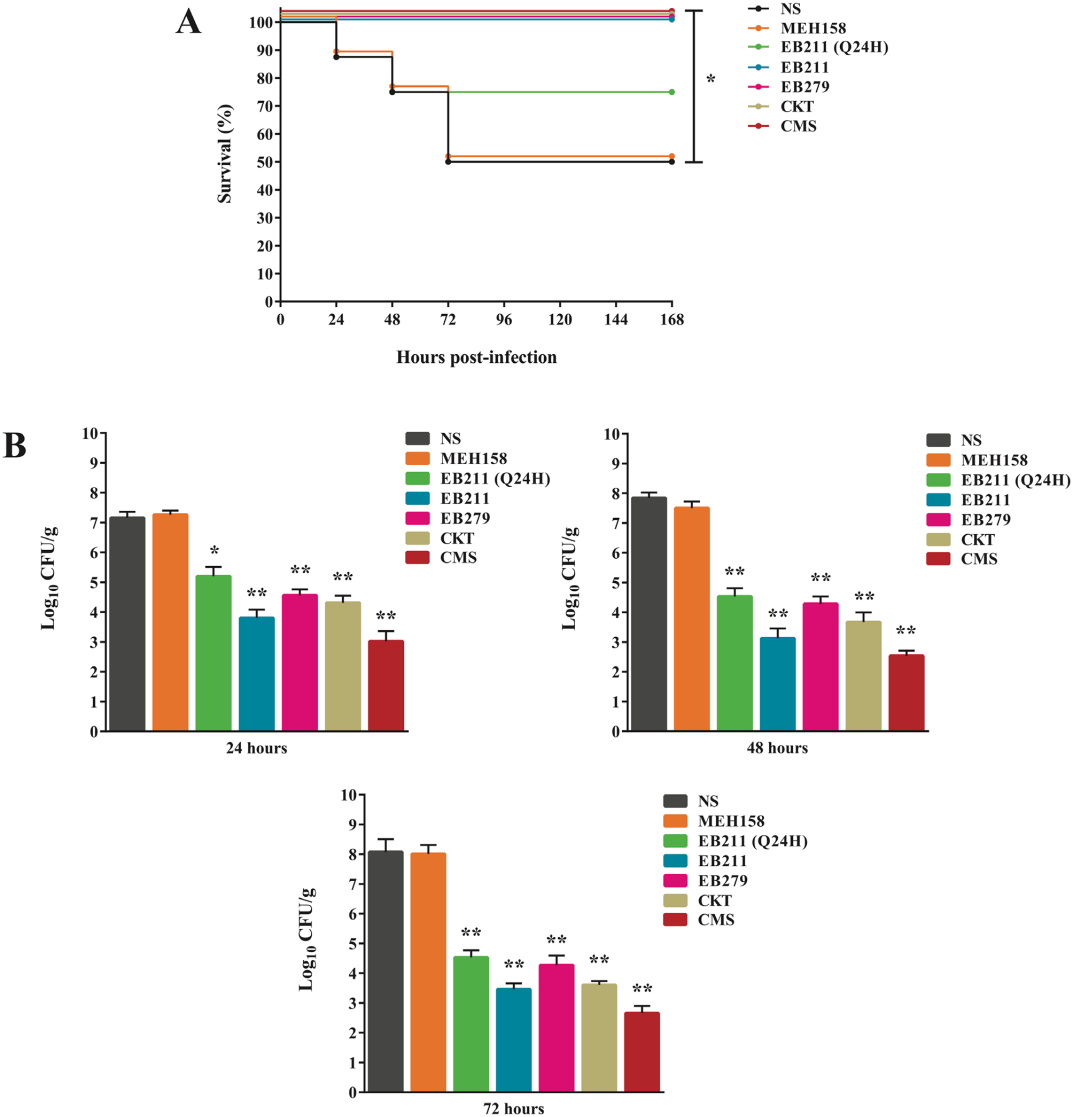
Administration of EB211 and EB279 led to an increased survival rate and a reduced bacterial burden in the lungs of immunocompromised mice with *A. baumannii* pneumonia. Immunocompromised mice were inoculated intranasally with XDR *A. baumannii* A.b.56 at a 50% lethal dose (LD_50_; 4×10^7^ CFU per mouse). Immunocompromised infected mice were administered intraperitoneally twice daily with EB211 (12 mg/kg), EB279 (12 mg/kg), a cocktail of the two scFvs (CKT; 6 mg/kg of each), an irrelevant scFv (MEH158; an *S. aureus*-specific scFv) (12 mg/kg), colistimethate sodium (CMS; 30 mg/kg), or normal saline (NS), or once daily with EB211 (12 mg/kg) (Q24H) two hours after inoculation for 72 h. (**A**) The survival rate of mice (n = 8) monitored daily for up to seven days after infection. The log-rank test was used to compare Kaplan-Meier survival curves. *p < 0.05 for EB211, EB279, CKT, or CMS versus NS. The results represent three independent experiments. (**B**) Bacterial burden in the lungs of immunocompromised infected mice (n = 6) administered with the scFvs, CMS, or NS after 24, 48, and 72 h of infection. The results represent the mean ± SEM of three independent experiments. *p < 0.05 for EB211 (Q24H) versus NS; **p < 0.01 for EB211, EB211 (Q24H), EB279, CKT, or CMS versus NS.

Twice daily treatment of immunocompromised mice, infected with *A. baumannii* A.b.56 at the 50% lethal dose (LD_50_; 4×10^7^ CFU per mouse), with EB211 (12 mg/kg), EB279 (12 mg/kg), a combination of the two scFvs (6 mg/kg of each), or CMS (30 mg/kg) for 72 h, resulted in 100% survival rate after seven days, while a 50% survival rate was observed in mice treated twice daily with NS or MEH158 (an irrelevant scFv; 12 mg/kg) (Fig. 2A). Furthermore, once daily treatment of immunocompromised infected mice with EB211 for 72 h increased the survival rate to 75% after seven days (Fig. 2A).

The administration of EB211, EB279, a cocktail of the two scFvs, or CMS at two hours post-infection led to a significant reduction in *A. baumannii* burden in the lungs of mice at 24, 48, and 72 h of infection compared to mice treated with NS or MEH158 (Fig. 2B). Despite a high bacterial load in the lungs of NS-treated mice, twice daily administration of EB211, EB279, a cocktail of the two scFvs, or CMS resulted in the highest bacterial load reduction in the lungs of mice at 72 h of infection (Fig. 2B).

**Fig. 3:**
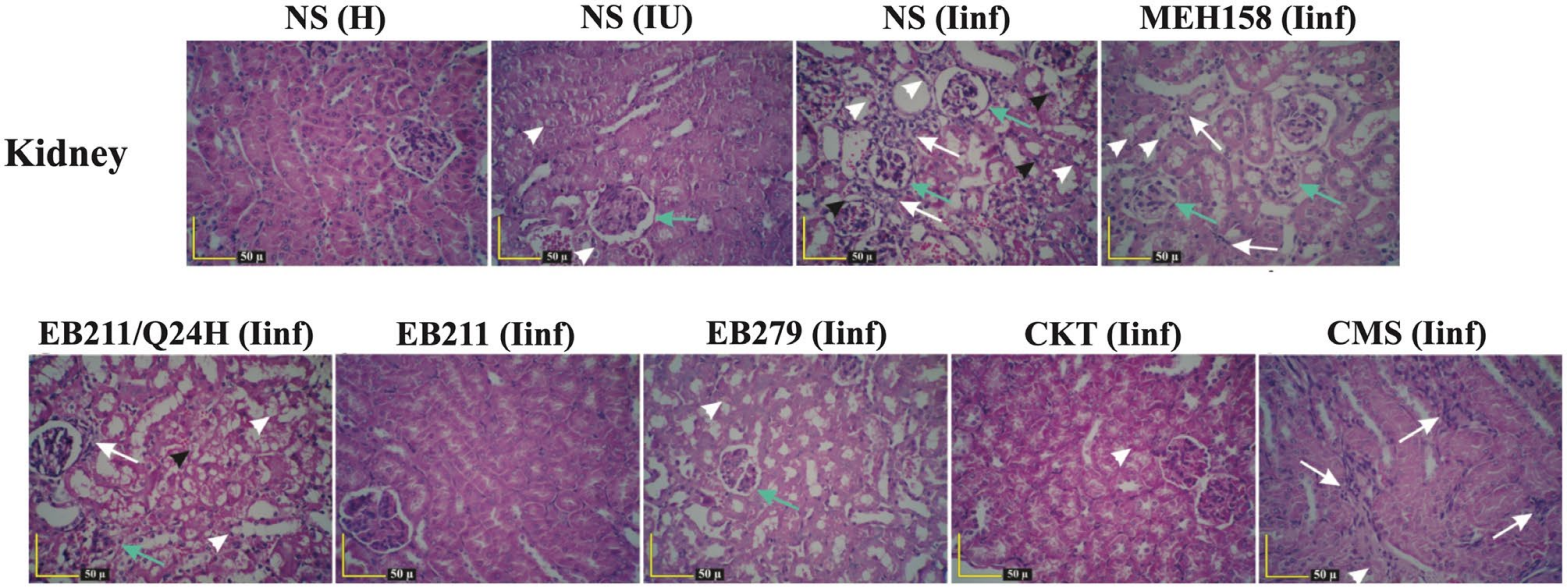
Administration of EB211 and EB279 prevented severe tissue damage in the kidneys of immunocompromised mice with *A. baumannii* pneumonia. Immunocompromised mice were inoculated intranasally with XDR *A. baumannii* A.b.56 at a 50% lethal dose (LD_50_; 4×10^7^ CFU per mouse). Immunocompromised infected mice were administered intraperitoneally twice daily with EB211 (12 mg/kg), EB279 (12 mg/kg), a cocktail of the two scFvs (CKT; 6 mg/kg of each), an irrelevant scFv (MEH158; an *S. aureus*-specific scFv) (12 mg/kg), colistimethate sodium (CMS; 30 mg/kg), or normal saline (NS), or once daily with EB211 (12 mg/kg) (Q24H) two hours after inoculation for 72 h. Healthy (H), immunocompromised uninfected (IU), and immunocompromised infected (Iinf) mice were killed at 72 h of infection, and the kidneys were collected. Tissue sections were stained with hematoxylin-eosin and microscopically evaluated for histopathological alterations. Tubular degeneration and necrosis, and infiltration of inflammatory cells (leukocytes) into the interstitial tissue surrounding the tubules in the cortex and medulla were the marked pathological symptoms in Iinf mice treated twice daily with NS, MEH158, or CMS, or once daily with EB211 (Q24H). After 72 h of infection, no severe pathological signs were detected in the kidneys of Iinf mice treated twice daily with EB211, EB279, or CKT for 72 h. Black arrowheads: tubular necrosis, Green arrows: diminished and distorted glomeruli, White arrows: infiltration of inflammatory cells (leukocytes), White arrowheads: cytoplasmic vacuolation and pale-staining plus fragmented cytoplasm

The histopathological examination of the kidneys, liver, and lungs from immunocompromised infected mice administered with EB211, EB279, or a cocktail of the two scFvs twice daily for 72 h demonstrated the significant protective effect of the scFvs against pneumonia caused by *A. baumannii* A.b.56. After 72 h of infection, tubular degeneration and necrosis, inflammatory cell infiltration (leukocytes) into the interstitial tissue surrounding the tubules in the cortex and medulla, and diminished and distorted glomeruli, and eosinophilic casts were observed in immunocompromised mice treated twice daily with NS, MEH158, or CMS, or once daily with EB211 (Fig. 3). In contrast, immunocompromised infected mice treated twice daily with EB211 and EB279 (alone and a cocktail of the two scFvs) showed no marked pathological symptoms, except cytoplasmic vacuolation and pale-staining plus diminished and distorted glomeruli, which might associate with cyclophosphamide (Fig. 3).

**Fig. 4:**
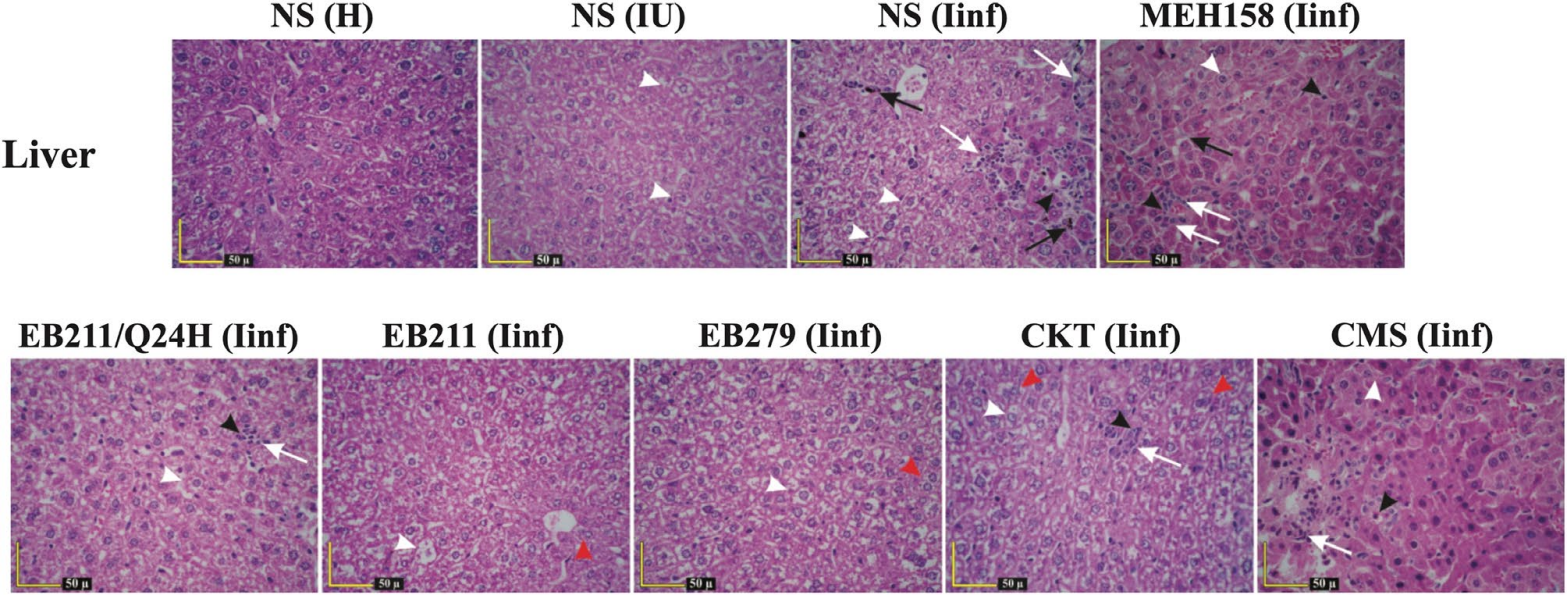
Administration of EB211 and EB279 prevented severe tissue damage in the liver of immunocompromised mice with *A. baumannii* pneumonia. Immunocompromised mice were inoculated intranasally with XDR *A. baumannii* A.b.56 at a 50% lethal dose (LD_50_; 4×10^7^ CFU per mouse). Immunocompromised infected mice were administered intraperitoneally twice daily with EB211 (12 mg/kg), EB279 (12 mg/kg), a cocktail of the two scFvs (CKT; 6 mg/kg of each), an irrelevant scFv (MEH158; an *S. aureus*-specific scFv) (12 mg/kg), colistimethate sodium (CMS; 30 mg/kg), or normal saline (NS), or once daily with EB211 (12 mg/kg) (Q24H) two hours after inoculation for 72 h. Healthy (H), immunocompromised uninfected (IU), and immunocompromised infected (Iinf) mice were killed at 72 h of infection, and the liver were collected. Tissue sections were stained with hematoxylin-eosin and microscopically evaluated for histopathological alterations. Bacterial communities and infiltration of inflammatory cells (neutrophils and mononuclear cells) along with spotty necrosis‌ were observed in the liver of Iinf mice treated twice daily with NS or MEH158. After 72 h of infection, no severe pathological signs were detected in the liver of Iinf mice treated twice daily with EB211, EB279, or CKT for 72 h. Black arrows: bacterial foci, Black arrowheads: necrosis of hepatocytes, Red arrowheads: bi-nucleated cells, White arrows: infiltration of inflammatory cells (mononuclear cells) along with spotty necrosis, White arrowheads: micro and macro vesicles

After 72 h, micro and macro vesicles were detected in the livers of immunocompromised uninfected and infected mice (Fig. 4). Bacterial communities and infiltration of inflammatory cells (neutrophils and mononuclear cells) along with spotty necrosis‌ were the marked pathological symptoms found in immunocompromised infected mice treated twice daily with NS or MEH158 (Fig. 4). Furthermore, mice treated twice daily with CMS or once daily with EB211 showed similar pathologic symptoms, except for bacterial foci. None of the immunocompromised infected mice treated twice daily with EB211, EB279, or a cocktail of the two scFvs showed severe pathological changes (Fig. 4).

**Fig. 5:**
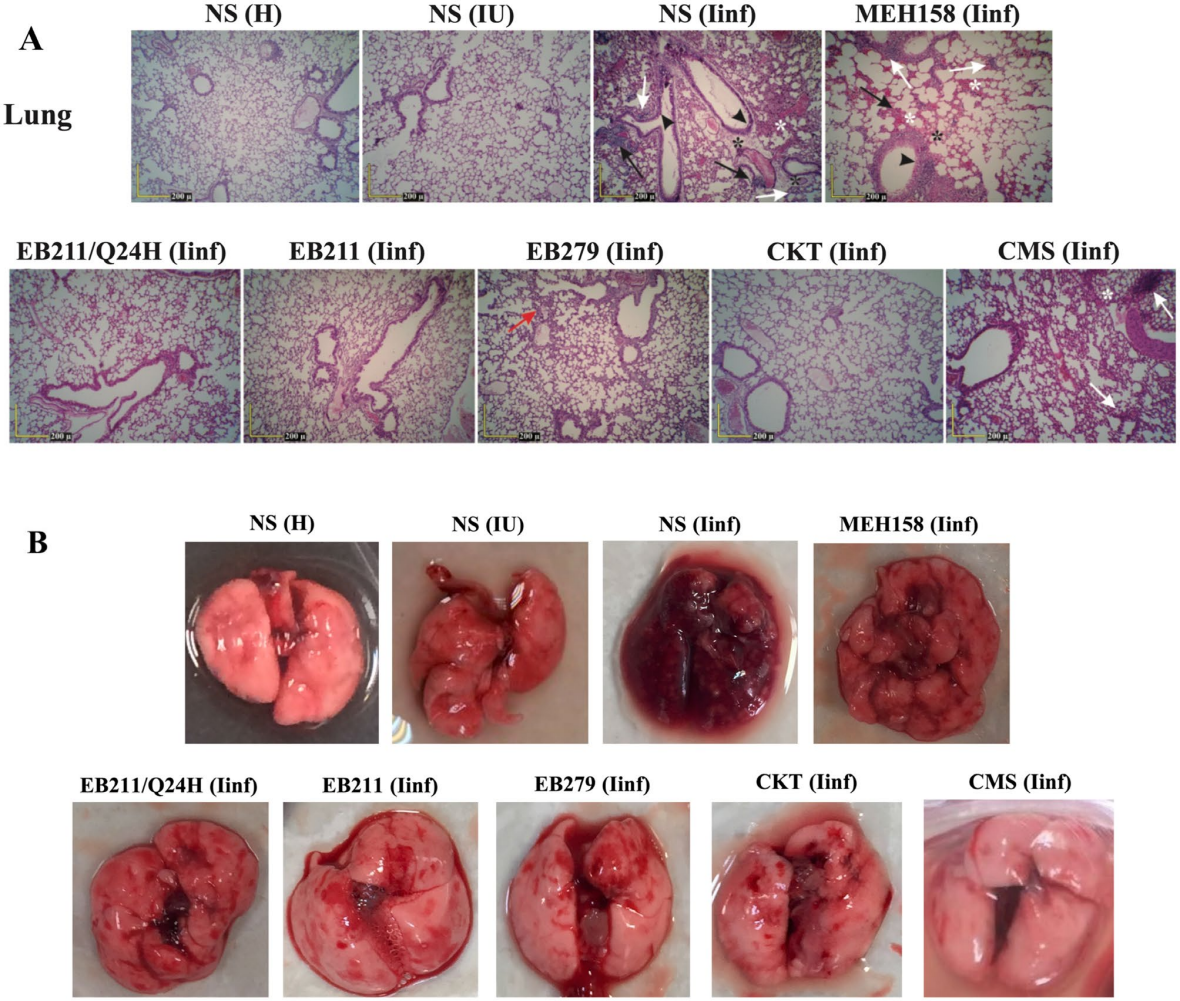
Administration of EB211 and EB279 prevented severe tissue damage in the lungs of immunocompromised mice with *A. baumannii* pneumonia. Immunocompromised mice were inoculated intranasally with XDR *A. baumannii* A.b.56 at a 50% lethal dose (4×10^7^ CFU per mouse). Immunocompromised infected mice were administered intraperitoneally twice daily with EB211 (12 mg/kg), EB279 (12 mg/kg), a cocktail of the two scFvs (CKT; 6 mg/kg of each), an irrelevant scFv (MEH158; an *S. aureus*-specific scFv) (12 mg/kg), colistimethate sodium (CMS; 30 mg/kg), or normal saline (NS), or once daily with EB211 (12 mg/kg) (Q24H) two hours after inoculation for 72 h. (**A**) and (**B**) Healthy (H), immunocompromised uninfected (IU), and immunocompromised infected (Iinf) mice were killed at 72 h of infection, and the lungs were collected. Tissue sections were stained with hematoxylin-eosin and microscopically evaluated for histopathological alterations. Bacterial communities in perivascular areas, infiltration of inflammatory cells (neutrophils and mononuclear cells) in the perivascular and peribronchial areas, parenchymal necrosis, necrosis of bronchial epithelial cells, edema of perivascular spaces, and hemorrhage in the alveoli and interstitium were detected in the lungs of Iinf mice treated twice daily with NS or MEH158. After 72 h of infection, no severe pathological signs were detected in the lungs of Iinf mice treated twice daily with EB211, EB279, or CKT for 72 h. Black arrows: bacterial foci, Black arrowheads: necrosis of bronchial epithelial cells, Black asterisks: edema of perivascular spaces, Red arrow: macrophage accumulation in the perivascular and peribronchial areas, White arrows: infiltration of inflammatory cells (neutrophils and mononuclear cells) in the perivascular and peribronchial areas, and parenchymal necrosis, White asterisks: areas of hemorrhage within alveoli and interstitium

After 72 h of infection, bacterial communities in perivascular areas, infiltration of inflammatory cells (neutrophils and mononuclear cells) in the perivascular and peribronchial areas, parenchymal necrosis, necrosis of bronchial epithelial cells, edema of perivascular spaces, and hemorrhage in the alveoli and interstitium were the significant pathological symptoms observed in the lungs of immunocompromised infected mice treated twice daily with NS or MEH158 (Fig. 5A and B). On the contrary, none of these pathological abnormalities were observed in the lungs of immunocompromised infected mice treated twice daily with EB211, EB279, or a cocktail of the two scFvs, or once daily with EB211 (Fig. 5A and B).

## Discussion

Up to now, several mAbs targeting *A. baumannii* have been developed, including anti-capsule mAbs [24, 42, 43], anti-OmpA and anti-Omp34 egg yolk antibodies (IgYs) [26], anti-OmpA mAbs [44], anti-siderophore-specific receptors (Iron Regulated Outer Membrane Proteins [IROMPs]) mAbs [45], and AR-401 (a fully human mAb; Aridis Pharmaceuticals, Inc.). There are controversial results about the efficacy of anti-capsule mAbs (13D6, C8, and 8E3 mAbs) against *A. baumannii* infection [24, 42, 43]. Two mAbs, 13D6 (an IgM specific to K1 capsular polysaccharide) [42] and C8 (an IgG1 specific to K4 capsular polysaccharide) [24], showed antibacterial activity in vitro and in vivo models of *A. baumannii* infections. In contrast, mAb 8E3, a mouse IgG3 specific to K2 capsular polysaccharide, exacerbated the disease in mice with pneumonia caused by *A. baumannii* strain AB899 [43]. Wang-Lin et al. indicated that antibody-dependent enhancement (ADE) of infection, previously reported for viruses such as Ebola virus [46], human immunodeficiency virus type 1 (HIV-1) [47, 48], dengue virus [49], and severe acute respiratory syndrome coronavirus 2 (SARS-CoV-2) [50], and “the sink effect” (binding of capsule-specific mAbs to shed capsule), led to the loss of therapeutic activity of mAb 8E3, followed by an increased bacterial burden in the blood, lungs, and spleen, and a low survival rate in infected mice [43]. Moreover, Jahangiri et al. reported a low survival rate in immunocompromised mice with *A. baumannii* pneumonia treated with a combination of anti-OmpA and Omp34 IgYs (particularly at a high dose) at four hours post-infection compared to immunocompromised infected mice receiving anti-OmpA IgY or anti-Omp34 IgY alone [26]. They hypothesized that some similarity between OmpA and Omp34 proteins with murine proteins and subsequent binding of anti-OmpA and anti-Omp34 IgYs to mouse proteins (as decoys) led to the ADE of *A. baumannii* infection in immunocompromised mice [26]. Of note, Goel et al. indicated that five mouse IgM mAbs targeting IROMPs, involved in the uptake of siderophore-iron complex, inhibited the growth of *A. baumannii* cells in vitro by two mechanisms, including the augmentation of opsonization and the interference with the biological activity of IROMPs (direct bactericidal activity) [45]. In any case, the ADE of bacterial infection might be observed with antibody-based therapy [21], exacerbating the disease in patients. In contrast, antibody fragments such as bactericidal scFvs [30, 35, 37, 38, 41], having remarkable features including target-specific binding, small size, great tissue penetration, phagocytes- and complement-independent antimicrobial activity (particularly in immunocompromised patients), and subsequently, no ADE effect (Fc-related unwanted events), can be substantial alternatives to conventional mAbs [28, 35, 51, 52]. Aiming to isolate bactericidal scFvs specific to *A. baumannii*, two scFvs, EB211 and EB279, with unique sequences and significant binding ability to *A. baumannii*, were identified in our previous study [40]. Of note, we demonstrated that EB211 and EB279 elicited an antibacterial effect against *A. baumannii* [40] possibly through the disruption in the outer membrane.

Off-target toxicity is one of the major causes impeding the evaluation of many therapeutics in clinical trials due to a group of various unwanted events in patients [53]. As abovementioned, Jahangiri et al. showed the similarity between peptides derived from OmpA and Omp34 proteins with some peptides of *Mus musculus* proteins (as decoys), resulting in the inefficacy of the combination anti-OmpA and anti-Omp34 IgYs in the treatment of mice with *A. baumannii* pneumonia [26]. In this regard, we investigated the off-target effect of both scFvs in vivo. The histopathological examination of the kidneys and liver of mice receiving EB211 (12 mg/kg), EB279 (12 mg/kg), or a cocktail of the two scFvs (6 mg/kg of each) twice daily for 72 h indicated no inflammation or tissue damage, confirming the safety of the scFvs in healthy mice.

In our previous study, EB211 (200 µg/ml), EB279 (200 µg/ml), and a cocktail of the two scFvs (100 µg/ml of each) showed significant growth inhibitory activity against an XDR *A. baumannii* strain in vitro (about 60%, 22%, and 60% reduction, respectively) [40]. Markedly, EB211 and EB279 alone did not exhibit a significant effect on the growth of *A. baumannii* at a concentration of 100 µg/ml; however, when these two scFvs were combined, acceptable results were obtained at the same concentration [40]. We hypothesized that combining two different antibodies at appropriate concentrations would be more advantageous than using a high concentration of one antibody. Since the latter may result in saturation of antibodies around the outer membranes of bacteria, leading to the presence of free antibodies that do not have the opportunity to bind and result in off-target binding in the host [54]. It is noteworthy that prior studies showed promising results when several antibodies were used in combination against pathogens such as *S. aureus* [30, 33]. In this regard, we investigated the therapeutic efficacy of a cocktail of the two scFvs (6 mg/kg of each) in addition to EB211 (12 mg/kg) alone and EB279 (12 mg/kg) alone in immunocompromised mice infected with an XDR *A. baumannii* strain. The administration dose of 12 mg/kg (for EB211 and EB279 alone) and 6 mg/kg (as a cocktail of two scFvs) was selected based on the concentration of the scFv which showed the highest activity against A. baumannii in vitro (alone, 200 µg/ml; as a cocktail, 100 µg/ml of each; [40]). EB211, EB279, and a cocktail of the two scFvs conferred complete protection against lethal pneumonia. Besides, immunocompromised infected mice administered twice daily with EB211, EB279, or a cocktail of the two scFvs for 72 h showed a lower bacterial load in the lungs and no marked histopathological abnormalities in the kidneys, liver, and lungs after 72 h of infection compared to immunocompromised infected mice administered twice daily with NS or an irrelevant scFv. A few studies assessed the protective efficacy of anti-*A. baumannii* mAbs in the murine model of pneumonia [24, 26, 43]. Nielsen et al. assessed the therapeutic efficacy of mAb C8, targeting the K4 capsular polysaccharide of *A. baumannii*, in a murine model of aspiration pneumonia [24]. Mice intratracheally infected with an XDR *A. baumannii* (strain HUMC1) received mAb C8 (5 µg/mouse/intravenously or 50 µg/mouse/intraperitoneally) immediately or four hours after infection [24]. At both doses and routes of administration, mAb C8 exhibited significant protective activity in infected mice after seven days. Moreover, the administration of mAb C8 (50 µg/mouse/intraperitoneally) led to a decreased bacterial burden in the blood and lungs and, no pathological symptoms in the lungs of infected mice compared to infected mice receiving an isotype control mAb (50 µg/mouse/intraperitoneally) at 24 h of infection [24]. Nonetheless, Wang-Lin et al. reported that the administration of mAb 8E3 (against the K2 capsular polysaccharide) (50 or 200 µg/g/intraperitoneally) immediately after intratracheal inoculation with *A. baumannii* strain AB899 caused increased disease severity (100% mortality at 48 h of infection) [43]. Additionally, a higher bacterial load in the blood, lungs, and spleen was observed in 8E3-treated mice than PBS-treated mice at 24 h of infection. Based on the findings, mAb 8E3 could not confer protection in infected mice [43]. In another study, Jahangiri et al. evaluated the therapeutic efficacy of a group of IgYs specific to OmpA, Omp34, and the whole inactivated cells of *A. baumannii* in immunocompromised mice intranasally inoculated with *A. baumannii* strain AbI101 [26]. All mice were intranasally administered with IgYs at four hours after inoculation. Based on the results, IgYs against OmpA and the whole inactivated cells had significant therapeutic activity and decreased the mortality by 25% in infected mice (75% survival) at eight days after inoculation [26]. In contrast, the combination of anti-OmpA and anti-Omp34 IgYs exacerbated the disease in mice due to the ADE effect, as previously mentioned [26].

## Conclusions

Two fully human anti-*A. baumannii* scFvs, EB211 and EB279, demonstrated no toxic activity in mice after receiving them (alone or combined). Of note, they exhibited significant therapeutic efficacy in immunocompromised mice with pneumonia caused by XDR *A. baumannii.* These data suggest that EB211 and EB279 may be effective against *A. baumannii* pneumonia.

## Methods

### Investigation of the toxic potential of EB211 and EB279 in healthy mice

To investigate the possible cytotoxic effects of EB211 and EB279, female C57BL/6 mice (groups of six, seven to eight-week-old), obtained from the Animal Laboratory of the Pasteur Institute of Iran, were injected intraperitoneally twice daily with EB211 (12 mg/kg), EB279 (12 mg/kg), or a cocktail of the two scFvs (6 mg/kg of each) for 72 h. Mice injected intraperitoneally twice daily with 30 mg/kg of CMS (Colistin is administered as the prodrug colistimethate sodium [CMS] [55–58]; Sigma-Aldrich, Saint Louis, USA) or NS for 72 h were used as the controls. All mice were sacrificed by intraperitoneal injection of an overdose (5 times the anesthetic dose; [59]) of ketamine and xylazine (Alfasan, Woerden, The Netherlands) at 72 h of infection. The kidneys and liver were aseptically removed and then fixed in 10% formalin for 24 h, followed by embedding in paraffin. Then, thin sections of tissues stained with hematoxylin-eosin were microscopically analyzed for histopathological changes.

### Evaluation of the protective efficacy of EB211 and EB279 in an immunocompromised mouse model of *A. baumannii* pneumonia

An immunocompromised mouse pneumonia model was established as described previously [60]. In brief, female C57BL/6 mice (groups of eight, seven to eight-week-old), acquired from the Animal Laboratory of the Pasteur Institute of Iran, were injected intraperitoneally with 150 mg/kg of cyclophosphamide (Sigma-Aldrich), four days and one day before intranasal challenge with *A. baumannii* [60]. On day zero, mice anesthetized by intraperitoneal injection of a combination of ketamine (100 mg/kg) and xylazine (10 mg/kg) were intranasally inoculated with XDR *A. baumannii* A.b.56 [40, 61] (at a 50% lethal dose [LD_50_]; 4×10^7^ CFU per mouse) [62]. Two hours after inoculation [63], mice were given EB211(12 mg/kg), EB279 (12 mg/kg), a cocktail of the two scFvs (6 mg/kg of each), or an irrelevant scFv (MEH158; an *S. aureus*-specific scFv) (12 mg/kg) intraperitoneally twice daily for 72 h. Additionally, in a different group, immunocompromised infected mice were treated intraperitoneally once daily with 12 mg/kg of EB211 for 72 h to investigate the dosing frequency on the success of treatment of pneumonia. The control mice were administered intraperitoneally twice daily with 30 mg/kg of CMS or NS for 72 h. Mice were monitored for disease symptoms [64, 65] and body weight [64], and the number of surviving animals was recorded daily for seven days [64].

Moreover, immunocompromised infected mice (groups of six), treated twice daily with EB211, EB279, a cocktail of the two scFvs, MEH158, CMS, or NS, or once daily with EB211 for 72 h, were euthanized with an overdose of ketamine and xylazine [59] after 24, 48, and 72 h of infection, and the lungs were harvested. The *A.baumannii* burden in the lungs was determined by plating the diluted tissue samples on Luria-Bertani agar (Merck, Darmstadt, Germany) containing imipenem (Sigma-Aldrich), followed by the enumeration of colonies grown after 18 h of incubation at 37 °C [60, 64, 66, 67].

Healthy mice, immunocompromised uninfected mice, and immunocompromised infected mice treated with EB211, EB279, a cocktail of the two scFvs, MEH158, CMS, or NS (groups of six) were sacrificed with an overdose of ketamine and xylazine [59] at 72 h after of infection, and the kidneys, liver, and lungs were harvested [64]. The thin sections of paraffin-embedded tissues were stained with hematoxylin-eosin and then investigated for histopathological alterations [64, 66–68].

### Statistical analyses

Statistical differences between the experimental groups were analyzed by Student’s *t*-test. The log-rank test was used to compare Kaplan-Meier survival curves. GraphPad Prism version 6 software (https://www.graphpad.com/) was used for all analyses, and differences were considered statistically significant at *p* values of < 0.05.

## Data Availability

All data generated or analyzed during this study are included in the manuscript. The datasets generated and/or analysed during the current study are available in the GenBank repository under accession numbers OQ970166 (EB211) and OQ970167 (EB279).
